# (*E*)-2-[(4-Ethoxy­phen­yl)imino­meth­yl]-4-methoxy­phenol

**DOI:** 10.1107/S1600536809040586

**Published:** 2009-10-10

**Authors:** Arzu Özek, Çiğdem Albayrak, Orhan Büyükgüngör

**Affiliations:** a Department of Physics, Ondokuz Mayıs University, TR-55139 Samsun, Turkey; b Faculty of Education, Sinop University, Sinop, Turkey

## Abstract

In the mol­ecule of the title compound, C_16_H_17_NO_3_, the aromatic rings are oriented at a dihedral angle of 29.25 (8)°. An intra­molecular O—H⋯N hydrogen bond results in the formation of a nearly planar [maximum deviation 0.034 (13) Å] six-membered ring, which is oriented at dihedral angles of 0.91 (1) and 28.91 (12)° with respect to the aromatic rings. The title mol­ecule is a phenol–imine tautomer, as evidenced by C—O, C—N and C—C bond lengths. In the crystal, mol­ecules are linked by inter­molecular C—H⋯O hydrogen bonds that generate *C*(8) chains.

## Related literature

For background to this study, see: Özek *et al.*, 2007 ▶. For related structures, see: Özek *et al.* (2009 ▶); Özek *et al.* (2008 ▶).
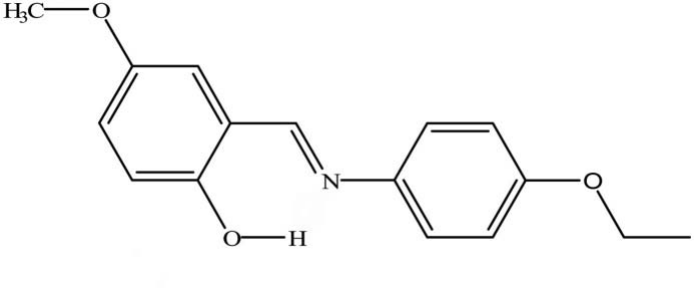


## Experimental

### 

#### Crystal data

C_16_H_17_NO_3_
*M* *_r_* = 271.31
Monoclinic, 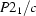

*a* = 14.8558 (7) Å
*b* = 13.7669 (7) Å
*c* = 6.9042 (3) Å
β = 90.287 (4)°
*V* = 1412.02 (11) Å^3^
*Z* = 4
Mo *K*α radiation
μ = 0.09 mm^−1^
*T* = 296 K
0.77 × 0.51 × 0.28 mm

#### Data collection

Stoe IPDS II diffractometer
Absorption correction: integration (*X-RED32*; Stoe & Cie, 2002 ▶) *T*_min_ = 0.943, *T*_max_ = 0.973
14704 measured reflections
2938 independent reflections
2014 reflections with *I* > 2σ(*I*)
*R*_int_ = 0.051

#### Refinement

*R*[*F* ^2^ > 2σ(*F* ^2^)] = 0.044
*wR*(*F* ^2^) = 0.123
*S* = 1.04
2938 reflections
250 parameters
All H-atom parameters refined
Δρ_max_ = 0.11 e Å^−3^
Δρ_min_ = −0.12 e Å^−3^

### 

Data collection: *X-AREA* (Stoe & Cie, 2002 ▶); cell refinement: *X-AREA*; data reduction: *X-RED32* (Stoe & Cie, 2002 ▶); program(s) used to solve structure: *SHELXS97* (Sheldrick, 2008 ▶); program(s) used to refine structure: *SHELXL97* (Sheldrick, 2008 ▶); molecular graphics: *ORTEP-3 for Windows* (Farrugia, 1997 ▶); software used to prepare material for publication: *WinGX* (Farrugia, 1999 ▶).

## Supplementary Material

Crystal structure: contains datablocks I, global. DOI: 10.1107/S1600536809040586/ez2189sup1.cif

Structure factors: contains datablocks I. DOI: 10.1107/S1600536809040586/ez2189Isup2.hkl

Additional supplementary materials:  crystallographic information; 3D view; checkCIF report

## Figures and Tables

**Table 1:** Hydrogen-bond geometry (Å, °)

*D*—H⋯*A*	*D*—H	H⋯*A*	*D*⋯*A*	*D*—H⋯*A*
O1—H1⋯N1	0.93 (3)	1.75 (3)	2.5962 (18)	149 (2)
C10—H10⋯O1^i^	0.965 (18)	2.571 (18)	3.3801 (19)	141.5 (13)

Symmetry code: (i) 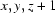.

**Fig. 1.**
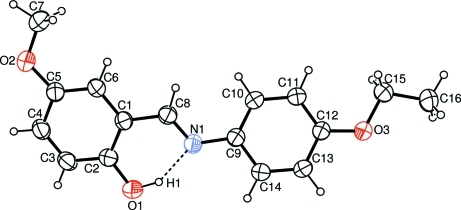
A view of (I), with the atom-numbering scheme and 30% probability displacement ellipsoids. The dashed line indicates the intramolecular hydrogen bond.

**Fig. 2.**
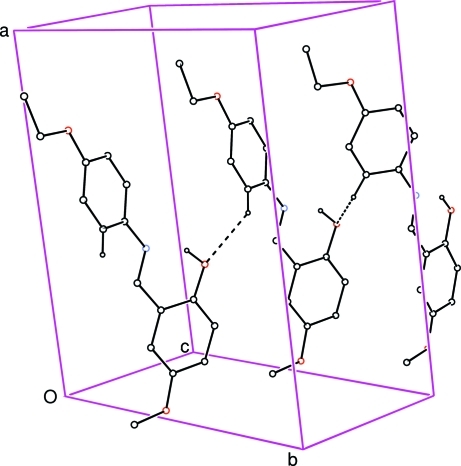
A partial packing view of (I), showing the formation of the C(8) chain through C—H···O hydrogen bonds (dashed lines). H atoms are represented as small spheres of arbitrary radii and H atoms not involved in hydrogen bonding have been omitted for clarity. Dashed lines indicate hydrogen bonds.

## supplementary crystallographic information

### Comment

The present work is part of a structural study of Schiff bases (Özek *et al.*, 2009; Özek *et al.*, 2008; Özek *et al.*, 2007) and we report here the structure of (*E*)-2-[(4-ethoxyphenylimino)methyl]-4-methoxyphenol, (I).

In general, *O*-hydroxy Schiff bases exhibit two possible tautomeric forms, the phenol-imine (or benzenoid) and keto-amine (or quinoid) forms. Depending on the tautomers, two types of intra-molecular hydrogen bonds are possible: O—H···N in benzenoid and N—H···O in quinoid tautomers. In the title compound the H atom is located on atom O1, thus the phenol-imine tautomer is favored over the keto-amine form, as indicated by the C2—O1, C8—N1, C1—C8 and C1—C2 bond lengths (Fig. 1 and Table 2). The O1—C2 bond length of 1.351 (2) Å indicates single-bond character, whereas the N1—C8 bond length of 1.277 (2) Å indicates a high degree of double-bond character. A similar result was observed in the X-ray crystal and computational structural study of (*E*)-2-[(2-chlorophenyl) iminomethyl]-4-methoxyphenol [C—O=1.357 (17) Å, C—N= 1.278 (17) Å, Özek *et al.*, 2008.

It is known that Schiff bases may exhibit thermochromism or photochromism, depending on the planarity or non-planarity of the molecule, respectively. Therefore, one can expect photochromic properties in (I) caused by non-planarity of the molecules; the dihedral angle between ring A (C1—C6) and ring B (C9—C14) is 29.25 (8) °. The intramolecular O—H···N hydrogen bond (Table 1) results in the formation of a nearly planar six-membered ring C (O1/H1/N1/C1/C2/C8), in which it is oriented with respect to rings A and B at dihedral angles of A/C= 0.91 (1) ° and B/C= 28.91 (12) °. It is thus coplanar with the adjacent ring A. It generates an S(6) ring motif. The O1···N1 distance of 2.5962 (18) Å is comparable to those observed for analogous hydrogen bonds in three (*E*)-2-[(bromophenyl)iminomethyl]-4-methoxyphenols [2.603 (2) Å, 2.638 (7) Å, 2.577 (4) Å; Özek *et al.*, 2007]. In the crystal structure, weak intermolecular C—H···O hydrogen bonds (Table 1) result in the formation of C(8) chains along the *c* axis (Fig. 2), which may play a role in the stabilization of the structure.

### Experimental

The compound (*E*)-2-[(4-ethoxyphenylimino)methyl]-4-methoxyphenol was prepared by refluxing a mixture of a solution containing 5-methoxysalicylaldehyde (0.5 g, 3.3 mmol) in 20 ml ethanol and a solution containing 4-ethoxyaniline (0.45 g, 3.3 mmol) in 20 ml ethanol. The reaction mixture was stirred for 1 h under reflux. Crystals of (*E*)-2-[(4-ethoxyphenylimino)methyl]-4- methoxyphenol suitable for X-ray analysis were obtained from ethanol by slow evaporation (yield % 75; m.p. 365–367 K).

### Refinement

All the H-atoms were found in difference-density maps, and refined freely. The C—H bond lengths are 0.90 (3)–1.06 (2) Å.

### Crystal data

**Table:**

C_16_H_17_NO_3_	*F*(000) = 576
*M**_r_* = 271.31	*D*_x_ = 1.276 Mg m^−^^3^
Monoclinic, *P*2_1_/*c*	Mo *K*α radiation, λ = 0.71073 Å
Hall symbol: -P 2ybc	Cell parameters from 14704 reflections
*a* = 14.8558 (7) Å	θ = 2.0–28.0°
*b* = 13.7669 (7) Å	µ = 0.09 mm^−^^1^
*c* = 6.9042 (3) Å	*T* = 296 K
β = 90.287 (4)°	Prism, brown
*V* = 1412.02 (11) Å^3^	0.77 × 0.51 × 0.28 mm
*Z* = 4	

### Data collection

**Table:**

Stoe IPDS II diffractometer	2938 independent reflections
Radiation source: fine-focus sealed tube	2014 reflections with *I* > 2σ(*I*)
plane graphite	*R*_int_ = 0.051
Detector resolution: 6.67 pixels mm^-1^	θ_max_ = 26.5°, θ_min_ = 2.0°
ω scans	*h* = −18→18
Absorption correction: integration (*X-RED32*; Stoe & Cie, 2002)	*k* = −17→17
*T*_min_ = 0.943, *T*_max_ = 0.973	*l* = −8→7
14704 measured reflections	

### Refinement

**Table:**

Refinement on *F*^2^	Secondary atom site location: difference Fourier map
Least-squares matrix: full	Hydrogen site location: inferred from neighbouring sites
*R*[*F*^2^ > 2σ(*F*^2^)] = 0.044	All H-atom parameters refined
*wR*(*F*^2^) = 0.123	*w* = 1/[σ^2^(*F*_o_^2^) + (0.0671*P*)^2^] where *P* = (*F*_o_^2^ + 2*F*_c_^2^)/3
*S* = 1.04	(Δ/σ)_max_ < 0.001
2938 reflections	Δρ_max_ = 0.11 e Å^−^^3^
250 parameters	Δρ_min_ = −0.12 e Å^−^^3^
0 restraints	Extinction correction: *SHELXL97* (Sheldrick, 2008), Fc^*^=kFc[1+0.001xFc^2^λ^3^/sin(2θ)]^-1/4^
Primary atom site location: structure-invariant direct methods	Extinction coefficient: 0.0043 (13)

### Special details

**Table:**

Experimental. 260 frames, detector distance = 100 mm
Geometry. All e.s.d.'s (except the e.s.d. in the dihedral angle between two l.s. planes) are estimated using the full covariance matrix. The cell e.s.d.'s are taken into account individually in the estimation of e.s.d.'s in distances, angles and torsion angles; correlations between e.s.d.'s in cell parameters are only used when they are defined by crystal symmetry. An approximate (isotropic) treatment of cell e.s.d.'s is used for estimating e.s.d.'s involving l.s. planes.
Refinement. Refinement of *F*^2^ against ALL reflections. The weighted *R*-factor *wR* and goodness of fit *S* are based on *F*^2^, conventional *R*-factors *R* are based on *F*, with *F* set to zero for negative *F*^2^. The threshold expression of *F*^2^ > σ(*F*^2^) is used only for calculating *R*-factors(gt) *etc*. and is not relevant to the choice of reflections for refinement. *R*-factors based on *F*^2^ are statistically about twice as large as those based on *F*, and *R*- factors based on ALL data will be even larger.

### Fractional atomic coordinates and isotropic or equivalent isotropic displacement parameters (Å^2^)

**Table:**

	*x*	*y*	*z*	*U*_iso_*/*U*_eq_	
C1	0.67303 (10)	0.38075 (10)	0.1954 (2)	0.0592 (4)	
C2	0.66776 (11)	0.36315 (11)	−0.0041 (2)	0.0644 (4)	
C3	0.74627 (12)	0.35280 (13)	−0.1091 (2)	0.0738 (5)	
C4	0.82841 (13)	0.35935 (13)	−0.0201 (2)	0.0743 (5)	
C5	0.83522 (11)	0.37538 (12)	0.1785 (2)	0.0677 (4)	
C6	0.75803 (10)	0.38680 (12)	0.2840 (2)	0.0633 (4)	
C7	0.93120 (15)	0.38888 (19)	0.4548 (3)	0.0868 (6)	
C8	0.59256 (11)	0.38970 (11)	0.3128 (2)	0.0635 (4)	
C9	0.43564 (10)	0.38370 (11)	0.3529 (2)	0.0587 (4)	
C10	0.43263 (11)	0.35185 (12)	0.5428 (2)	0.0663 (4)	
C11	0.35242 (11)	0.34958 (13)	0.6444 (2)	0.0669 (4)	
C12	0.27310 (10)	0.37865 (10)	0.5544 (2)	0.0593 (4)	
C13	0.27591 (11)	0.41068 (12)	0.3634 (2)	0.0658 (4)	
C14	0.35554 (10)	0.41219 (12)	0.2639 (2)	0.0647 (4)	
C15	0.18545 (13)	0.35600 (18)	0.8412 (3)	0.0808 (5)	
C16	0.08889 (15)	0.3588 (2)	0.8994 (4)	0.1001 (7)	
N1	0.51409 (8)	0.38318 (9)	0.23737 (18)	0.0634 (3)	
O1	0.58802 (9)	0.35510 (10)	−0.09793 (18)	0.0830 (4)	
O2	0.92111 (8)	0.37741 (11)	0.25288 (18)	0.0904 (4)	
O3	0.19015 (7)	0.37712 (8)	0.63865 (15)	0.0709 (3)	
H1	0.5439 (17)	0.3668 (16)	−0.006 (3)	0.116 (8)*	
H3	0.7423 (13)	0.3381 (14)	−0.243 (3)	0.100 (6)*	
H4	0.8816 (14)	0.3471 (14)	−0.091 (3)	0.093 (6)*	
H6	0.7613 (11)	0.3976 (13)	0.421 (3)	0.083 (5)*	
H7A	0.9038 (14)	0.4477 (18)	0.503 (3)	0.105 (7)*	
H7B	0.8999 (13)	0.3335 (15)	0.528 (3)	0.093 (6)*	
H7C	0.9976 (15)	0.3864 (13)	0.473 (3)	0.096 (6)*	
H8	0.6036 (11)	0.4006 (12)	0.449 (3)	0.080 (5)*	
H10	0.4858 (12)	0.3266 (13)	0.606 (2)	0.082 (5)*	
H11	0.3521 (11)	0.3218 (13)	0.777 (3)	0.084 (5)*	
H13	0.2221 (11)	0.4322 (12)	0.307 (2)	0.073 (5)*	
H14	0.3585 (10)	0.4324 (13)	0.130 (2)	0.077 (5)*	
H15A	0.2218 (13)	0.4035 (14)	0.913 (3)	0.090 (6)*	
H15B	0.2105 (13)	0.2887 (16)	0.862 (3)	0.112 (7)*	
H16A	0.0524 (19)	0.304 (2)	0.825 (4)	0.158 (11)*	
H16B	0.0618 (16)	0.4162 (19)	0.873 (4)	0.128 (9)*	
H16C	0.0829 (14)	0.3476 (15)	1.038 (4)	0.105 (7)*	

### Atomic displacement parameters (Å^2^)

**Table:**

	*U*^11^	*U*^22^	*U*^33^	*U*^12^	*U*^13^	*U*^23^
C1	0.0603 (8)	0.0579 (8)	0.0594 (8)	0.0004 (6)	0.0039 (6)	−0.0015 (6)
C2	0.0680 (9)	0.0663 (10)	0.0589 (8)	−0.0002 (7)	−0.0019 (7)	0.0026 (7)
C3	0.0824 (12)	0.0850 (12)	0.0542 (9)	0.0049 (8)	0.0085 (8)	0.0022 (8)
C4	0.0714 (11)	0.0849 (12)	0.0666 (10)	0.0082 (8)	0.0157 (8)	0.0059 (8)
C5	0.0598 (9)	0.0752 (10)	0.0682 (9)	0.0016 (7)	0.0055 (7)	0.0066 (7)
C6	0.0619 (9)	0.0718 (10)	0.0561 (8)	−0.0011 (7)	0.0043 (7)	−0.0025 (7)
C7	0.0665 (12)	0.1075 (17)	0.0864 (13)	−0.0070 (11)	−0.0097 (9)	−0.0061 (12)
C8	0.0634 (9)	0.0665 (10)	0.0605 (9)	0.0000 (7)	0.0005 (7)	−0.0065 (7)
C9	0.0577 (8)	0.0580 (8)	0.0604 (8)	0.0005 (6)	−0.0006 (6)	−0.0042 (6)
C10	0.0574 (8)	0.0769 (10)	0.0645 (9)	0.0066 (7)	−0.0065 (7)	0.0038 (7)
C11	0.0627 (9)	0.0768 (10)	0.0610 (9)	0.0051 (7)	−0.0026 (7)	0.0078 (8)
C12	0.0546 (8)	0.0601 (9)	0.0633 (8)	0.0009 (6)	0.0002 (6)	−0.0028 (7)
C13	0.0580 (9)	0.0769 (10)	0.0625 (9)	0.0070 (7)	−0.0078 (7)	0.0016 (7)
C14	0.0651 (9)	0.0737 (10)	0.0554 (8)	0.0031 (7)	−0.0026 (7)	0.0020 (7)
C15	0.0702 (11)	0.1014 (15)	0.0707 (11)	0.0031 (10)	0.0062 (8)	0.0190 (10)
C16	0.0712 (13)	0.139 (2)	0.0902 (15)	0.0020 (13)	0.0187 (11)	0.0247 (15)
N1	0.0595 (8)	0.0655 (8)	0.0651 (7)	−0.0001 (6)	0.0016 (6)	−0.0029 (6)
O1	0.0742 (8)	0.1130 (10)	0.0616 (7)	−0.0007 (6)	−0.0088 (6)	−0.0047 (6)
O2	0.0576 (7)	0.1344 (12)	0.0794 (8)	0.0014 (6)	0.0047 (6)	0.0062 (7)
O3	0.0564 (6)	0.0901 (8)	0.0661 (6)	0.0021 (5)	0.0011 (5)	0.0055 (5)

### Geometric parameters (Å, °)

**Table:**

C1—C2	1.401 (2)	C9—C14	1.393 (2)
C1—C6	1.403 (2)	C9—N1	1.4154 (19)
C1—C8	1.453 (2)	C10—C11	1.386 (2)
C2—O1	1.3518 (19)	C10—H10	0.965 (18)
C2—C3	1.384 (2)	C11—C12	1.388 (2)
C3—C4	1.367 (3)	C11—H11	0.990 (17)
C3—H3	0.94 (2)	C12—O3	1.3653 (18)
C4—C5	1.392 (2)	C12—C13	1.392 (2)
C4—H4	0.95 (2)	C13—C14	1.371 (2)
C5—C6	1.371 (2)	C13—H13	0.936 (17)
C5—O2	1.373 (2)	C14—H14	0.966 (17)
C6—H6	0.957 (18)	C15—O3	1.431 (2)
C7—O2	1.410 (2)	C15—C16	1.492 (3)
C7—H7A	0.97 (2)	C15—H15A	0.98 (2)
C7—H7B	1.03 (2)	C15—H15B	1.01 (2)
C7—H7C	0.99 (2)	C16—H16A	1.06 (3)
C8—N1	1.277 (2)	C16—H16B	0.90 (3)
C8—H8	0.963 (18)	C16—H16C	0.98 (2)
C9—C10	1.384 (2)	O1—H1	0.93 (3)
			
C2—C1—C6	119.01 (14)	C9—C10—H10	120.8 (10)
C2—C1—C8	121.41 (14)	C11—C10—H10	117.9 (10)
C6—C1—C8	119.55 (13)	C10—C11—C12	119.84 (15)
O1—C2—C3	118.65 (14)	C10—C11—H11	118.9 (10)
O1—C2—C1	122.00 (14)	C12—C11—H11	121.1 (10)
C3—C2—C1	119.35 (15)	O3—C12—C11	124.83 (14)
C4—C3—C2	120.70 (16)	O3—C12—C13	116.10 (13)
C4—C3—H3	120.3 (12)	C11—C12—C13	119.06 (14)
C2—C3—H3	119.0 (12)	C14—C13—C12	120.63 (15)
C3—C4—C5	120.92 (16)	C14—C13—H13	121.6 (10)
C3—C4—H4	120.2 (12)	C12—C13—H13	117.7 (10)
C5—C4—H4	118.7 (12)	C13—C14—C9	120.83 (15)
C6—C5—O2	125.23 (15)	C13—C14—H14	121.8 (9)
C6—C5—C4	119.02 (16)	C9—C14—H14	117.4 (9)
O2—C5—C4	115.75 (14)	O3—C15—C16	108.04 (16)
C5—C6—C1	120.99 (15)	O3—C15—H15A	109.3 (11)
C5—C6—H6	120.3 (10)	C16—C15—H15A	111.9 (11)
C1—C6—H6	118.7 (10)	O3—C15—H15B	107.7 (12)
O2—C7—H7A	113.0 (12)	C16—C15—H15B	109.9 (12)
O2—C7—H7B	110.8 (11)	H15A—C15—H15B	109.9 (17)
H7A—C7—H7B	105.2 (17)	C15—C16—H16A	109.9 (15)
O2—C7—H7C	103.0 (11)	C15—C16—H16B	113.3 (16)
H7A—C7—H7C	113.8 (16)	H16A—C16—H16B	107 (2)
H7B—C7—H7C	111.3 (15)	C15—C16—H16C	110.6 (13)
N1—C8—C1	121.24 (14)	H16A—C16—H16C	108 (2)
N1—C8—H8	123.9 (10)	H16B—C16—H16C	107 (2)
C1—C8—H8	114.9 (10)	C8—N1—C9	121.49 (13)
C10—C9—C14	118.40 (14)	C2—O1—H1	106.1 (14)
C10—C9—N1	124.26 (13)	C5—O2—C7	117.78 (14)
C14—C9—N1	117.20 (13)	C12—O3—C15	117.91 (12)
C9—C10—C11	121.23 (14)		
			
C6—C1—C2—O1	179.03 (14)	C9—C10—C11—C12	0.7 (3)
C8—C1—C2—O1	1.0 (2)	C10—C11—C12—O3	178.28 (15)
C6—C1—C2—C3	−0.4 (2)	C10—C11—C12—C13	−0.7 (2)
C8—C1—C2—C3	−178.42 (15)	O3—C12—C13—C14	−178.05 (15)
O1—C2—C3—C4	−179.32 (15)	C11—C12—C13—C14	1.0 (2)
C1—C2—C3—C4	0.2 (3)	C12—C13—C14—C9	−1.3 (3)
C2—C3—C4—C5	0.8 (3)	C10—C9—C14—C13	1.3 (2)
C3—C4—C5—C6	−1.5 (3)	N1—C9—C14—C13	177.16 (14)
C3—C4—C5—O2	177.91 (16)	C1—C8—N1—C9	174.64 (13)
O2—C5—C6—C1	−178.13 (15)	C10—C9—N1—C8	−27.7 (2)
C4—C5—C6—C1	1.2 (2)	C14—C9—N1—C8	156.79 (15)
C2—C1—C6—C5	−0.3 (2)	C6—C5—O2—C7	2.3 (3)
C8—C1—C6—C5	177.78 (14)	C4—C5—O2—C7	−177.08 (18)
C2—C1—C8—N1	−1.0 (2)	C11—C12—O3—C15	8.4 (2)
C6—C1—C8—N1	−179.00 (14)	C13—C12—O3—C15	−172.62 (16)
C14—C9—C10—C11	−1.0 (2)	C16—C15—O3—C12	179.78 (18)
N1—C9—C10—C11	−176.53 (15)		

### Hydrogen-bond geometry (Å, °)

**Table:**

*D*—H···*A*	*D*—H	H···*A*	*D*···*A*	*D*—H···*A*
O1—H1···N1	0.93 (3)	1.75 (3)	2.5962 (18)	149 (2)
C10—H10···O1^i^	0.965 (18)	2.571 (18)	3.3801 (19)	141.5 (13)

Symmetry codes: (i) *x*, *y*, *z*+1.
